# Determinants of gain modulation enabled by short-term depression at an inhibitory cerebellar synapse

**DOI:** 10.1186/1471-2202-15-S1-O11

**Published:** 2014-07-21

**Authors:** Dimitris Bampasakis, Reinoud Maex, Neil Davey, Volker Steuber

**Affiliations:** 1 Science and Technology Research Institute, University of Hertfordshire, Hatfield AL10 9AB, UK; 2 Department of Cognitive Sciences, École Normale Supérieure, Paris 75005, France

## 

Neurons adapt rapidly the slope, also known as gain, of their input-output function to time-varying conditions. Gain modulation is a prominent mechanism in many brain processes, such as auditory processing and attention scaling of orientation tuning curves. It is known to amplify neuronal signals, prevent firing saturation, and play a key role in coordinate transformation [1].

Synaptic short-term depression (STD) at the excitatory synapse from mossy fibres (MFs) to granule cells in the cerebellum has previously been found to introduce a gain change, and enhance inhibition-mediated gain modulation [2]. Similar results were discovered for STD at the inhibitory synapse from Purkinje cells (PCs) to cerebellar nucleus (CN) neurons, where STD modulates gain and enhances excitation-mediated gain modulation [3]. In both cases – whether STD is applied at the excitatory or inhibitory synapse, respectively – the non-linearity introduced by STD in the relationship between input firing rate and average conductance, was found to underlie the effects of STD.

We use a multi-compartmental model of a cerebellar nucleus neuron [4] to understand how STD at an inhibitory synapse can add a multiplicative component in the transformation performed by excitatory input. To do so, we use input from PCs, applied at an inhibitory synapse with STD, and excitatory input from MFs, while changing the level of STD by manipulating the presynaptic release probability (R) [5]. We find that gain modulation resulting from the introduction of STD increases with the extent of depression. To further our understanding, we investigate the effects of STD using synchronous input, regular input, and their combination. We find that the multiplicative component introduced by STD remains, but varies in value for different input conditions. Moreover, we present a detailed analysis of how a non-linear mapping between input spike rate and synaptic conductance can result in multiplicative operations.
